# Editorial: Circuits plasticity in neurodegenerative disorders: targeting mood disorders

**DOI:** 10.3389/fncir.2025.1592657

**Published:** 2025-04-15

**Authors:** Yvan M. Vachez, Philippe Huot, Robin Magnard

**Affiliations:** ^1^Inserm, U1216, Univ. Grenoble Alpes, Grenoble Institut Neurosciences, Grenoble, France; ^2^Neurodegenerative Disease Group, Montreal Neurological Institute-Hospital (The Neuro), Montreal, QC, Canada; ^3^Department of Neurology and Neurosurgery, McGill University, Montreal, QC, Canada; ^4^Movement Disorder Clinic, Division of Neurology, Department of Neuroscience, McGill University Health Centre, Montreal, QC, Canada; ^5^Department of Psychological and Brain Sciences, Krieger School of Arts and Sciences, Johns Hopkins University, Baltimore, MD, United States

**Keywords:** mood disorder, Parkinson's disease, addiction, dyskinesia, globus pallidus, zona incerta, thalamus, calcium channel

Neurodegenerative disorders affect millions of people worldwide, presenting not only motor impairments but also severe mood and cognitive disturbances. Current treatments remain largely symptomatic and non-specific, sometimes even exacerbating psychiatric complications. Emerging evidence indicates that maladaptive plasticity in neural circuits could be responsible for both motor and non-motor symptoms. This Research Topic, “*Circuits Plasticity in Neurodegenerative Disorders: Targeting Mood Disorders*,” gathers studies employing neuroimaging, chemogenetics, and molecular techniques, along with opinion reviews to elucidate the shared neurobiological mechanisms linking psychiatric symptoms and neurodegeneration.

In this Research Topic, Wilt et al. focused on the zona incerta (ZI), a small, understudied sub-thalamic structure now recognized as a heterogeneous hub. Initially investigated for its role in ingestive behaviors and basic survival, numerous investigations have then established the ZI's contributions in motor control, particularly in Parkinson's disease (PD), with deep brain stimulation improving motor symptoms when applied not only to sub-thalamic nucleus but also to ZI. Wilt et al. synthesize recent findings highlighting the contribution of ZI to reward motivation and hypothesizing its potential involvement in addiction. These evidences include the ZI's connectivity, or its neuronal populations reminiscent of the substantia nigra pars compacta (SNc) and ventral tegmental area composition, with the notable presence of dopaminergic neurons. Thus, the ZI may be integrated into neural circuits underlying aberrant reward processes, including addiction, although its precise role in encoding reward prediction errors or driving compulsive behaviors for instance remains unclear. The authors suggest that targeting ZI circuits could open novel therapeutic avenues for treating addiction-related disorders.

Expanding on connectivity, Cirillo et al. revealed a direct anatomical pathway between the SNc and the thalamus in young healthy humans. Using multi-shell high-angular resolution diffusion imaging (MS-HARDI) tractography on 10 subjects (5 males, 5 females; ages 25–30), they precisely segmented the SNc and thalamus from high-resolution 3D T1 images. Their probabilistic tractography isolated streamlines traversing the SNc and terminating in the thalamus, while excluding fibers to other regions. Approximately 12% of SNc streamlines are directly connected with the thalamus in both hemispheres, a finding further supported by normative PET analysis of dopamine receptor densities showing similar profiles in both regions. Despite limitations such as imaging resolution and potential false positives, reproducibility across two MS-HARDI schemes reinforces this nigro-thalamic pathway, expanding our understanding of dopaminergic regulation beyond the classic nigro-striatal circuit.

In a neuromodulation study, Lipari et al. investigated whether selectively inhibiting serotonin (5-HT) projections from the dorsal raphe nucleus could improve L-DOPA-induced dyskinesia (LID) and Parkinson's disease-associated psychosis (PDAP) in a rat model of PD. Using TPH2-Cre Long Evans rats with bilateral 6-hydroxydopamine lesions of the medial forebrain bundle, they infused a Cre-dependent inhibitory DREADD into the dorsal raphe and administered the designer drug Compound 21 (C21) during chronic L-DOPA treatment. Motor performance was measured via a stepping test, and abnormal involuntary movements (AIMs) were quantified, with pre-pulse inhibition (PPI) assessing sensorimotor gating. Acute inhibition of dorsal raphe 5-HT neurons with C21 significantly reduced LID without affecting sensorimotor function, suggesting that abnormal 5-HT activity contributes to dyskinetic movements.

Addressing molecular mechanisms of depressive behavior, He et al. explored how modulation of L-type Ca^2^^+^ channel activity and DNA methylation interact in an lipopolysaccharide (LPS)-induced depression model in male C57BL/6J mice. Chronic LPS injections reduced body weight, sucrose preference, and increased immobility in the forced swim test, accompanied by decreased Dnmt3a expression in the hippocampal dentate gyrus. While the antidepressant Venlafaxine reversed these deficits and normalized inflammation markers (GFAP), the L-type Ca^2^^+^ channel agonist BAY K 8644 alone only partially improved depressive-like behavior without restoring Dnmt3a levels. Notably, combining BAY K 8644 with L-methionine significantly enhanced behavioral improvements, suggesting a synergistic interaction between Ca^2^^+^ channel modulation and DNA methylation in regulating neurodegenerative-induced mood disorders.

Finally, Dong et al. review the connectivity and functionality of the external segment of the globus pallidus (GPe), once considered a simple relay in the BG but now considered as a heterogeneous structure. The GPe comprises predominantly GABAergic neurons, divided into prototypic and arkypallidal subtypes, and a minority of cholinergic cells. The review details extensive afferents from the striatum, sub-thalamic nucleus, cortex, thalamus, and brainstem, and diverse efferents to BG output nuclei and the cortex. In PD, dopaminergic loss disrupts GPe firing patterns and increases β oscillations, contributing to both motor and non-motor symptoms. The authors emphasize the need to investigate distinct GPe neurons subpopulation to improve diagnosis and therapeutic interventions for PD.

All together, these articles underscore the evolution of our comprehension of neurodegenerative disorders, driven by maladaptive circuit plasticity across diverse neural networks. By integrating advanced techniques, the research highlights novel circuit-based therapeutic targets that may pave the way for more precise and effective treatments for mood disorders.

